# Controlled atmosphere treatment reduces fruit surface pitting by improving antioxidant capacity and modulating membrane lipid metabolism of refrigerated sweet cherries

**DOI:** 10.1016/j.fochx.2025.103240

**Published:** 2025-11-04

**Authors:** Qifeng Zhao, Yingjian Qi, Feng Wang, Haixia Yang, Qingzhen Yang, Xiaoping Zhang

**Affiliations:** a Pomology Institute, Shanxi Agricultural University, Taiyuan, Shanxi Province 030801, PR China; b Department of Life Sciences, Yuncheng University, Yuncheng, Shanxi Province 044000, PR China; c Featured Fruit Quality Control and Application Laboratory, Department of Life Science, Yuncheng University, Yuncheng 044000, PR China; d College of Food Science and Engineering, Shanxi Agricultural University, Taiyuan, Shanxi Province 030801, PR China

**Keywords:** Sweet cherry, Pitting, Controlled atmosphere, Antioxidant capacity, Membrane lipid metabolism

## Abstract

Sweet cherries are susceptible to surface pitting during low-temperature storage. This study examined the effects of a controlled-atmosphere treatment (3 % O_2_ + 10 % CO_2_ + 87 % N_2_) on pitting, antioxidant capacity, and membrane lipid metabolism in sweet cherry stored at 0 ± 0.5 °C. The controlled-atmosphere treatment.

effectively suppressed pitting and decay while preserving fruit quality. It enhanced the activities and gene expression levels of catalase, superoxide dismutase, glutathione reductase, and ascorbate peroxidase, while also increased ascorbic acid and glutathione contents, thereby promoting the removal of reactive oxygen species. Simultaneously, the treatment downregulated the expression and activity of lipoxygenase and phospholipase D, resulting in increased fatty acid unsaturation and improved preservation of cell membrane integrity and function. These findings indicate that a controlled-atmosphere treatment can improve the antioxidant capacity, mitigate membrane lipid peroxidation, and thereby effectively reduce surface pitting in refrigerated sweet cherries.

## Highlights

• Controlled-atmosphere treatment inhibited pitting and maintained quality of sweet cherry.
• Controlled-atmosphere treatment increased the antioxidant enzyme activities and relative expression levels.
• Controlled-atmosphere treatment enhanced antioxidant substance content.
• Controlled-atmosphere treatment slowed membrane lipid peroxidation and maintained high fatty acid unsaturation.

## 1 Introduction

Sweet cherry (*Prunus avium* L.) is particularly popular with consumers for its attractive colour, sweet and sour flavour, rich nutritional value, and strong antioxidant properties (Pan et al., 2025). However, owing to its thin and juicy skin, soft texture, and the high ambient temperatures encountered during harvesting, the fruit is highly susceptible to browning, softening, and decay (Afonso et al., 2023). Although cold storage effectively delays senescence and quality degeneration, prolonged exposure to low temperatures can induce chilling injury, manifested as surface pitting. Surface pitting is a physiological disorder of fruits, characterized by one or more localized depressions on the epidermal surface, resulting from the collapse of subepidermal cells, and can manifest anywhere on the fruit skin surface (Ponce et al., 2023). Fruits with the occurrence of pitting not only reduce consumers appeal, but also accelerates fruit decay, thereby compromising edibility and commercial value. Therefore, alleviating fruit pitting remains a critical challenges for the sweet cherry industry.

Low-temperature injury is closely associated with impaired cell membrane function and structure (Kuang et al., 2023). Increased storage time leads to the accumulation of reactive oxygen species (ROS) including hydrogen peroxide (H_2_O_2_) and superoxide anion (O_2_^.-^), which aggravates the process of oxidation (Yang et al., 2024). This oxidative damage further results in the accumulation of reactive substances such as malondialdehyde (MDA), damaging the cell membrane structure and function. In addition, exposure to low temperatures accelerates lipid degradation and peroxidation, altering the phospholipid composition of cell membranes. These changes are typically reflected by an increase in saturated fatty acids content and a decrease in unsaturated fatty acids content, which compromise membrane structural integrity and functionality. Consequently, such alterations contribute to chilling-induced fruit disorders, including surface pitting (Song et al., 2023). Therefore, regulating ROS scavenging capacity and membrane lipid metabolism in fruits during low-temperature storage is very essential for mitigating pitting disorders. At present, numerous methods, including gibberellin application (Einhorn et al., 2013), H_2_S treatment (Zhi & Dong, 2018), and ultraviolet irradiation (Michailidis et al., 2019) treatments, have been reported to alleviate surface pitting in sweet cherry. However, further efforts are warranted to explore better approaches for addressing this problem.

Controlled-atmosphere technology adjusts the ratio of O_2_, CO_2_, and N_2_ gases in the storage environment to delay fruit senescence and prolong the storage period. Controlled-atmosphere technology is safe and environmentally friendly, can better maintain fruit flavour and nutritional value (Li et al., 2019), and has been widely used in the post-harvest storage and transportation of fruits, such as pear (Lucas et al., 2024), apricot (Dorostkar et al., 2022), and avocado (Fuentealba et al., 2022). Studies have shown that appropriate controlled-atmosphere treatment during storage can considerably improve the antioxidant capacity of peach (Dong et al., 2022) and apple (Mditshwa et al., 2017). Additionally, it maintains cell membrane stability, and improve fruit storage quality. Liu et al. (2022) showed that controlled-atmosphere treatment during storage reduced the enzyme activity and downregulated the expression of lipoxygenase (LOX) in peach, increased fatty acid unsaturation, and effectively alleviated the symptoms of physiological disorders in fruits. Collectively, these studies indicate that a controlled-atmosphere plays a key role in modulating post-harvest storage quality and physiological and metabolic disorders in fruits. However, previous studies on controlled-atmosphere storage of sweet cherry have primarily focused on browning (Yang et al., 2019) and microbial control (Serradilla et al., 2013), whereas its effects on surface pitting have not yet been reported. Therefore, we used the ‘Sunny’ sweet cherry to investigate the impact of controlled-atmosphere on the capacity of antioxidants capacity, storage quality and surface pitting, and membrane lipid metabolism. This study aims to provide a theoretical basis and technical reference for the application of controlled-atmosphere treatment in the storage and transportation of post-harvest sweet cherries.

## 2 Materials and methods

### 2.1 Materials and treatments

‘Sunny’ sweet cherries were obtained from the sweet cherry demonstration base in Xia County, Yuncheng City. Fruits were hand-picked at 90 % maturity (transverse diameter > 26 mm, per fruit weight > 10 g, bright red colour). They were transported to the laboratory within 0.5 h of picking and were placed in cold storage at 5 °C for 3 h to dissipate the field heat.

Fruits uniform in colour and size and free from defects or mechanical damage were selected for the experiment. They were randomly divided into two groups, each including three replicates (10 kg of fruit per replicate). The first group of fruits was stored in a controlled-atmosphere chamber with a relative humidity of 90 %–95 % and an air composition of 3 % O_2_ + 10 % CO_2_ + 87 % N_2_ at 0 ± 0.5 °C (screened on the basis of preliminary experiments). The other group of fruits was stored at 0 ± 0.5 °C, with a relative humidity of 90 %–95 %, using mechanical cold storage as the control (CK). Samples were collected at 7-day intervals, with 75 fruits taken from each replicate. A total of 45 fruits were used to determine fruit colour, firmness, titratable acidity, and soluble solids contents. In addition, the fruits were cored and cut into small pieces, immediately frozen in liquid nitrogen, and stored at −80 °C in an ultra-low temperature freezer for the determination of O_2_^.-^, H_2_O_2_, MDA, antioxidant enzyme activities, fatty acid content, and other related indicators. Other 30 fruits were kept at 25 °C for 72 h to simulate shelf-life conditions, after which the pitting rate, pitting index, and decay rate were determined. Each treatment included three replicates, and the experiment was performed twice. As similar results were obtained in both experiments; data from one representative experiment are presented.

### 2.2 Evaluation of fruit pitting

A total of 30 fruits were selected to determine the pitting rate, pitting index, and decay rate. The fruit pitting rate was calculated according to Eq. (1):

(1) $$\text{Pitting rate}/\%=\frac{\text{number of pitting fruit}}{\text{total number of fruit}}\times 100$$

The pitting index was calculated based on the method developed by Nian et al. (2022). Based on the the degree of surface pitting, pitting was divided into four grades: 1 = mild pitting (pitting area ≤ 10 %), 2 = moderate pitting (11 % ≤ pitting area ≤ 20 %), 3 = severe pitting (21 % ≤ pitting area ≤ 30 %). 4 = extremely severe pitting (pitting area > 31 %). The pitting index was calculated according to Eq. (2):

(2) $$\text{pitting index}/\%=\frac{\sum \left(\text{Pitting grade}\times \text{The number of pitting of this grade}\right)}{\text{Total number of fruit}\times 4}\times 100$$

The fruit decay rate was calculated according to Eq. (3):

(3) $$\text{decay rate}/\%=\frac{\text{Number of decayed fruit}}{\text{Total number of fruit}}\times 100$$

### 2.3 Measurement of fruit colour, firmness, titratable acidity, and soluble solids contents

The firmness of fruit was detected using texture analyzer (TMS-Pro; Food Technology Corporation, Sterling, VA, USA). After removing the peel from both sides of the fruit at the equatorial portion, a cylindrical probe measuring 3 mm in diameter was utilized to penetrate the fruit at 1 mm/s to a depth of 3 mm, and the force was measured in N. Fruit colour was measured using a CR-400 colour-difference meter (Konica Minolta, Tokyo, Japan) to assess the brightness (L*), colour saturation (C), and hue angle (h°). Soluble solids content (SSC, %) was determined using a digital display saccharometer (model 3810; AtagoPAL-1, Saitama, Japan). Titratable acidity (TA) was determined according to Ding et al. (2024), and expressed as a percentage of malic acid (%).

### 2.4 Determination of O_2_^.-^, H_2_O_2_, MDA contents and electrolyte leakage

O_2_^.-^ (μg/g) and H_2_O_2_ (mmol/g) contents were determined according to Yang et al. (2021). MDA (μmol/g) and electrolyte leakage were tested in accordance with the approach of Wang et al. (2019).

### 2.5 Determination of antioxidant-related enzyme activity

The activities of superoxide dismutase (SOD) and catalase (CAT) were determined according to Han et al. (2023). An inhibitor of SOD with one active unit (U) is defined as the amount of inhibitor required per gram of fruit pulp to inhibit 50 % of the aziridinium blue tetrazolium photoresponse per minute, with the unit expressed as U/g. Definition of CAT activity is a change in absorbance of 0.01 per minute per gram of pulp in U/g. The activities of ascorbate peroxidase (APX) and glutathione reductase (GR) were assayed following the approach of Xu et al. (2021). The enzyme activity unit (U) of APX was defined as U/g, equivalent to a decrease in absorbance of 0.01 per gram per minute. The enzyme activity unit (U) of GR, represented as U/g, is equivalent to a change in absorbance of 0.01 per minute per gram of pulp reaction system.

### 2.6 Determination of antioxidant substances

The contents of ascorbic acid (AsA) contents and reduced glutathione (GSH) in sweet cherry were determined according to Huang et al. (2023), reported in mg/g and μmol/g, respectively.

### 2.7 Determination of LOX and phospholipase D (PLD) activities

LOX activity was assayed in accordance with the description by Yang et al. (2024). The unit of enzyme activity (U), represented as U/g, was determined to be a variation in absorbance of 0.001 per gram of pulp per minute. PLD activity was identified via the approach of Shuai et al. (2024), and the unit of enzyme activity (U), represented as U/g, was defined as a variation in the absorbance of 0.001 per gram of pulp per minute.

### 2.8 Fatty acid content determination

The fatty acids in sweet cherry were extracted and quantified according to Dai et al. (2021) using an HP-FFAP polyethylene glycol TPA capillary column (0.2 mm × 25 m × 0.33 μm; 7890–5975; Agilent, Santa Clara, CA, USA), For two minutes, GC–MS analysis was conducted at an initial temperature of 160 °C. Two minutes later, the temperature was increased to 200 °C at a rate of 15 °C per minute. Subsequently, the temperature was raised to 230 °C at a rate of 10 °C per minute and was maintained for five minutes. The shunt ratio and flow rate were 2:1 and 1 mL/min, respectively, and the contents of each component in each sample were calculated as follows:

$$\text{Fatty acid unsaturation}=\frac{\text{unsaturated fatty acid relative content}}{\text{saturated fatty acid relative content}.}$$

### 2.9 Determination of relative expression levels of key enzyme genes

Total RNA from sweet cherry was extracted according to Song et al. (2022), and a PrimeScript™ II 1st Strand cDNA Synthesis Kit (Takara Bio Inc., Shiga, Japan) was employed for the reverse transcription of RNA into cDNA. β-actin, a sweet cherry actin gene, was chosen as the internal reference gene. Primers specific for the relevant genes were designed using the NCBI website and Primer Premier software (version 5.0; Premier Biosoft, Palo Alto, CA, USA), and the primers were produced by Jiangsu Shengong Bioengineering Company (Shanghai, China). Table 1 displays the primer sequences. In accordance with the manufacturer's instructions, quantitative real-time fluorescence PCR was performed using a Magic SYBR Mixture Kit (Jiangsu CoWin Biotech Co., Ltd., Suzhou, China), and the 2-∆∆Ct approach was applied to determine the relative expression of genes.

**Table 1:** Target gene-specific primer sequence.

Gene	Primer Sequence (5′ → 3′)	Primer Sequence
*PaSOD1*	F GTCTAAGCAGAAATGGCGGG	255
	R TGGCGCTCTGTAATTTGACG	
*PaCAT*	F CTCATACTGGTCTCAGGCAGA	233
	R TGTGAACTACAAGAACGACTGC	
*PaAPX*	F ATTTCTACCAGTTGGCCGGA	254
	R TGTCCAGGGTCCCTCAAATC	
*PaGR*	F ATTGATGGTGAGGTGAGCCA	264
	R GGCATCCTCGATTTCACCAC	
*PaLOX5*	F TAACAGGGGTACTGTGGCTG	246
	R CTGCTTTTCTTGGGTGCCTT	
*PaPLD1*	F ATCACAGGAAGGTGTGAGGG	242
	R CAGCATCCACAAGCACTGTT	
β-actin	F AGCAACTGGGATGACATGGA	248
	R ACACCATCACCAGAGTCCAG	

### 2.10 Statistical analysis

The SPSS 27 software (SPSS Inc., Chicago, IL, USA) was utilized for statistical analysis of the test data, with results expressed as the mean ± standard error (SE). The significance of the differences was tested through the independent-samples Student's t-test (homogeneity of variance was confirmed by Levene's test, *p* > 0.05), with a significance level set at 0.05. Correlation analysis and principal component analysis (PCA) were implemented with Origin 2021 software. Statistical significance was defined as *p* < 0.05 and *p* < 0.01.

## 3 Results

### 3.1 Effects of controlled-atmosphere treatment on the pitting rate, pitting index, and decay rate in sweet cherry fruits

**Fig. 1:**
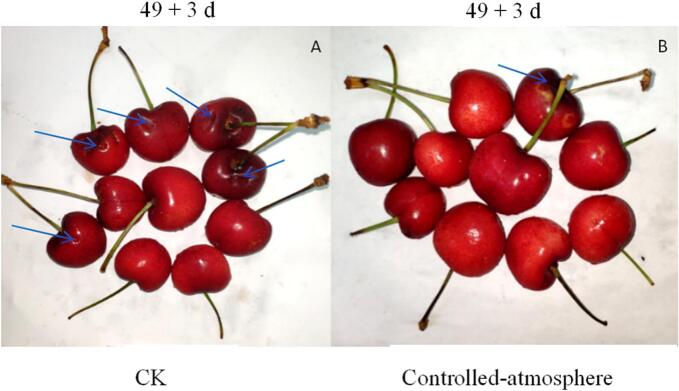
Impact of controlled-atmosphere treatment on appearance quality. Blue arrow: pitting symptom. (For interpretation of the references to colour in this figure legend, the reader is referred to the web version of this article.)

**Fig. 2:**
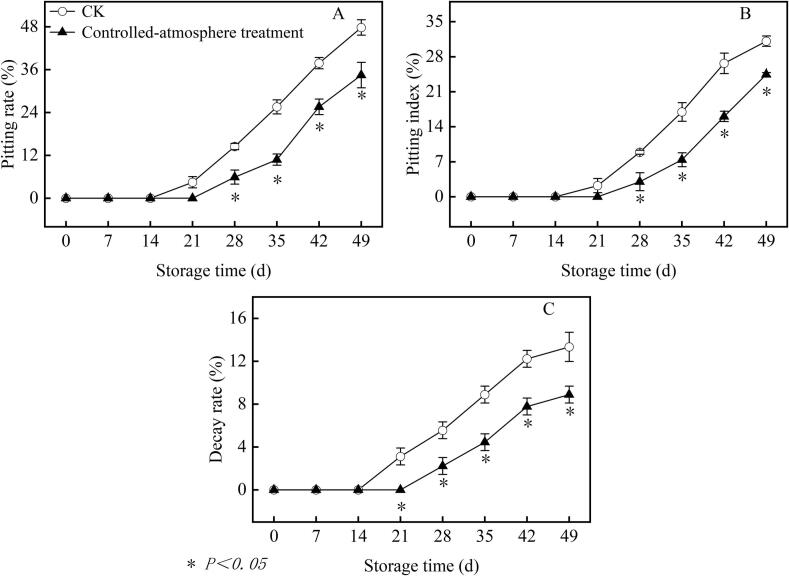
Impact of controlled-atmosphere treatment on the pitting rate (A), pitting index (B), and decay rate (C) of sweet cherry fruits. Data are expressed as mean ± standard error (SE, *n* = 3). * indicate significant differences at *P < 0.05*, according to the *t*-test.

Pitting appeared as one or more irregular indentations measuring 4–8 mm on the fruit surface. It could occur anywhere on the fruit, though it generally develops at the shoulder near the stem end or in the equatorial region of the fruit. Controlled-atmosphere treatment delayed and inhibited the occurrence of pitting in sweet cherries (Fig. 1 and Fig. 2). The fruits in the CK group developed pitting on day 21, while those in the controlled-atmosphere treatment group developed pitting on day 28 (Fig. 2A and B). Compared with the CK group, the controlled-atmosphere treatment markedly suppressed fruit pitting rate and pitting index. From day 28 to the conclusion of storage, the controlled-atmosphere group had a fruit pitting index and pitting rate that were 21.44 % and 27.92 % lower on average than the CK group (*P < 0.05*).

On the 21st day of storage for the CK group along with the 28th day of storage for the controlled-atmosphere group, respectively, fruit deteriorated (Fig. 2C). From day 21 to the end of storage, the fruit decay rate in the controlled-atmosphere treatment group was on average 45.82 % lower than CK group from the 21st day until the conclusion of storage (*P < 0.05*).

### 3.2 Effects of controlled-atmosphere treatment on storage quality in sweet cherry fruits

**Fig. 3:**
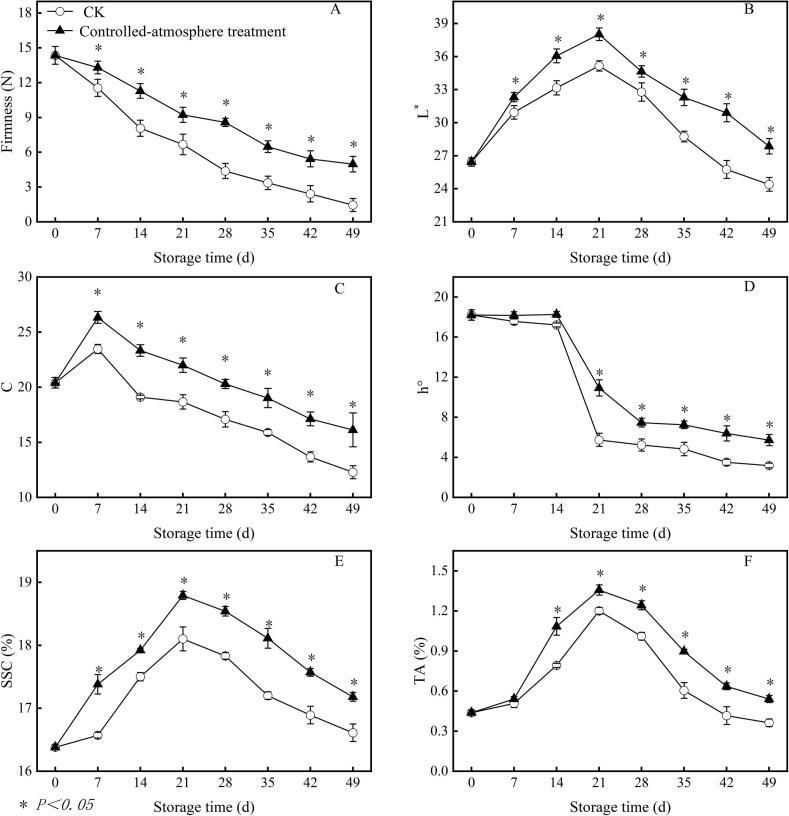
Impact of controlled-atmosphere treatment on the firmness (A), L* (B), C (C), h° (D), soluble solids content (E), and titratable acid content (F) of sweet cherry fruits. Data are expressed as mean ± SE (n = 3). * indicate significant differences at *P < 0.05*, according to the t-test.

Compared with the CK group, the controlled-atmosphere treatment maintained higher fruit firmness, L*, C, and SSC throughout storage. From day 7 to the conclusion of storage, these parameters in the controlled-atmosphere group were on average 56.38 %, 10.02 %, 20.05 %, and 3.81 % higher than those in the CK group (*P < 0.05*) (Figs. 3A, B, C, and E).

Significant differences (*P < 0.05* of h° values between the two groups were observed day 21 to the end of storage. At the conclusion of the storage period, fruits under controlled-atmosphere treatment exhibited 78.87 % higher h° values compared to the CK group (Fig. 3D).

In contrast to CK group, the controlled-atmosphere group showed consistently highe TA values during storage (Fig. 3F), with 31.51 % higher TA content compared to the CK group from day 14 to the conclusion of storage (*P < 0.05*).

### 3.3 Effects of controlled-atmosphere treatment on O_2_^.-^, H_2_O_2_, MDA content, and electrolyte leakage in sweet cherry fruits

**Fig. 4:**
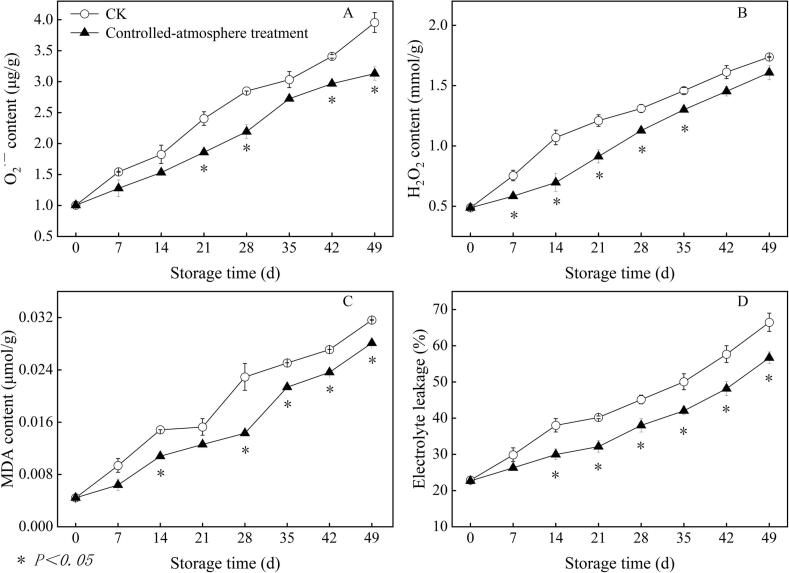
The impact of controlled-atmosphere treatment on O_2_^.-^ (A), H_2_O_2_ (B), MDA content (C) and electrolyte leakage (D) of sweet cherry fruits. Data are represented as mean ± standard error (n = 3). * indicate significant differences at *P < 0.05*, according to the t-test.

Remarkable differences were observed in O_2_^.-^ content between both groups from day 21 to the conclusion of storage, with the exception of day 35 of storage (*P < 0.05*) (Fig. 4A). The controlled-atmosphere group had a 20.76 % lower content of O_2_^.-^ than that in the CK group at the conclusion of storage.

Fruits in the controlled-atmosphere group had a lower H_2_O_2_ concentration after storage than did fruits in the CK group (Fig. 4B). The controlled-atmosphere group had a lower mean H_2_O_2_ content of 20.69 % than that in the CK group between days 7 and 35 of storage (*P < 0.05*).

In the controlled-atmosphere treatment group, the content of MDA was considerably lower than that in the CK group (*P < 0.05*) (Fig. 4C). The controlled-atmosphere group had 15.76 % lower content of MDA content than that in the CK group during the course of the storage period.

Electrolyte leakage was decreased by the controlled-atmosphere treatment from day 14 to the conclusion of the storage period, with an average decrease of 16.96 % compared to the CK group (*P < 0.05*) (Fig. 4D).

### 3.4 Effects of controlled-atmosphere treatment on the activities of CAT, SOD, GR and APX in sweet cherry fruits

**Fig. 5:**
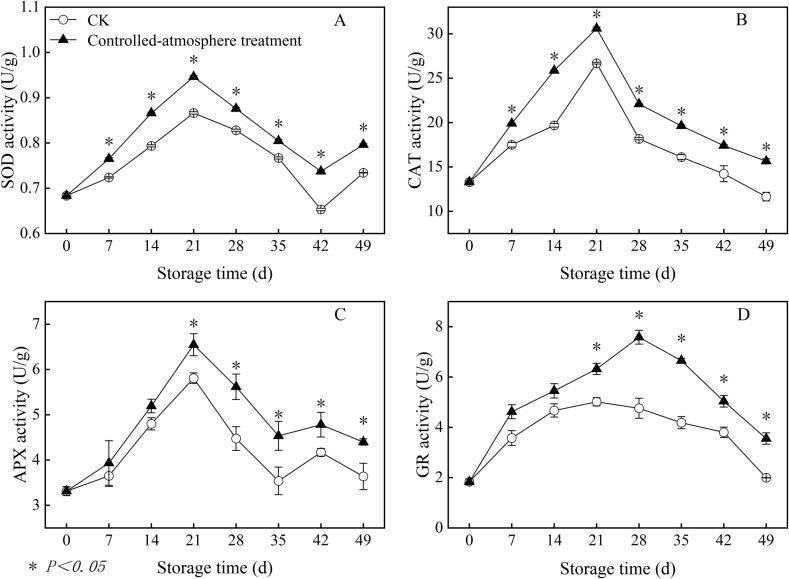
Impact of controlled-atmosphere treatment on the SOD (A), CAT (B), APX (C), and GR (D) activities in sweet cherry fruits. Data are expressed as mean ± SE (n = 3). * indicate significant differences at *P < 0.05*, according to the t-test.

On day 21 of storage, the sweet cherry fruits achieved their peak SOD activity (Fig. 5A). At this point, the SOD activity of the CK group was 9.20 % lower than that of the controlled-atmosphere treatment group. From day 14 to the conclusion of the storage period, a noticeable difference was observed between the two groups (*P < 0.05*).

Fig. 5B displayed that the CAT activity of sweet cherry fruits peaked on the day 21 of storage (Fig. 5B). From day 7 to the conclusion of the storage period, the differences between the two groups were highly significant (*P < 0.05*). At the conclusion of the storage period, the controlled-atmosphere group exhibited 34.33 % higher CAT activity compared to that of the CK group.

The APX activity of both groups was the highest on day 21 of storage, with that of the controlled-atmosphere group being 12.59 % higher than that of the CK group (Fig. 5C). The difference between both groups was significant from day 21 to the conclusion of storage (*P < 0.05*).

The peak value was reached on the day 28 of storage in the controlled-atmosphere group and on the day 21 in the CK group (Fig. 5D). The controlled-atmosphere group exhibited 47.59 % higher GR activity compared to that of the CK group from day 21 to the conclusion of storage (*P < 0.05*).

### 3.5 Effects of controlled-atmosphere treatment on the relative expression levels of *PaSOD1*, *PaCAT*, *PaAPX*, and *PaGR* in sweet cherry fruits

**Fig. 6:**
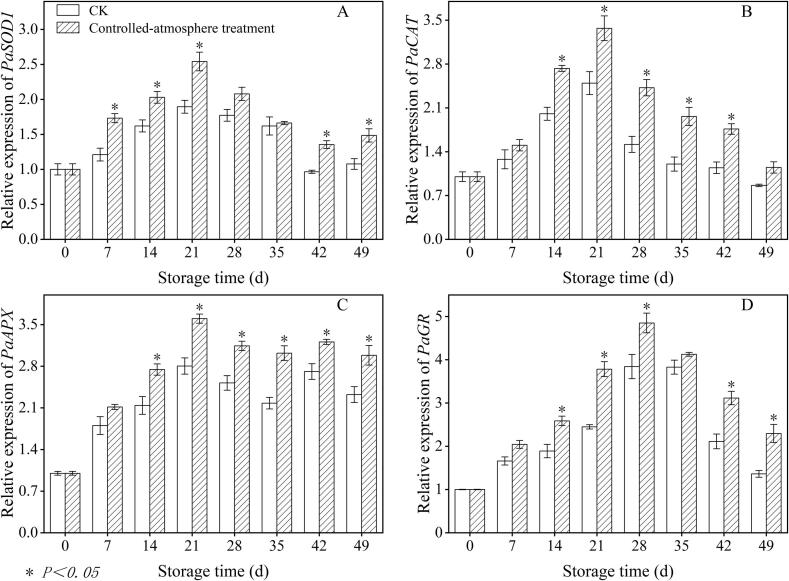
Impact of controlled-atmosphere treatment on the relative expression levels of *PaSOD1* (A), *PaCAT* (B), *PaAPX* (C), and *PaGR* (D) in sweet cherry fruits. Data are shown as mean ± SE (n = 3). * indicate significant differences at *P < 0.05*, according to the t-test.

Controlled-atmosphere treatment promoted *PaSOD1* expression, with its relative expression peaking on the day 21 of storage (Fig. 6A). Throughout the storage period, the controlled-atmosphere group exhibited 25.18 % higher relative expression of *PaSOD1* compared to that of the CK group (*P < 0.05*).

As shown in Fig. 6B, *PaCAT* relative expression levels peaked on the day 21 of storage. The controlled-atmosphere group exhibited 46.71 % higher *PaCAT* relative expression than that of the CK group from day 14 to day 42 of storage (*P < 0.05*).

During storage, controlled-atmosphere treatment promoted *PaAPX* expression. From day 14 until the end of storage period, the controlled-atmosphere group exhibited 27.35 % higher relative *PaAPX* expression levels compared to those of the CK group (*P < 0.05*) (Fig. 6C).

Fig. 6D showed that the relative expression of *PaGR* in sweet cherry peaked on the day 28 of storage. From the day 14 until the conclusion of storage, with the exception of day 35, the relative expression level of *PaGR* was significantly different between the two groups (*P < 0.05*), with the levels in the controlled-atmosphere group were 42.92 % higher than those of the CK group.

### 3.6 Effects of controlled-atmosphere treatment on AsA and GSH contents in sweet cherry fruits

**Fig. 7:**
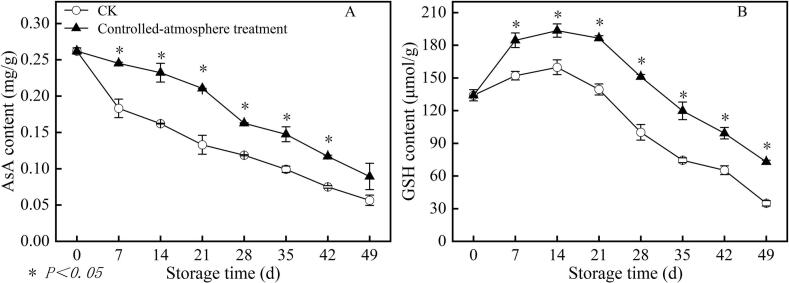
Impact of controlled-atmosphere treatment on the content of AsA (A) and GSH (B) in sweet cherry fruits. Data are expressed as mean ± SE (n = 3). * indicate significant differences at *P < 0.05*, according to the t-test.

From the day 7 to the day 42 of storage, the controlled-atmosphere treatment maintained higher AsA content, with levels 44.59 % than that in the CK group (*P < 0.05*) (Fig. 7A).

In both groups, the maximal value was attained on day 14 of storage. The content of GSH in the controlled-atmosphere treatment group was 38.79 % more than that in the CK group from day 7 to the conclusion of storage (*P < 0.05*).

### 3.7 Effects of controlled-atmosphere treatment on LOX and PLD activities in sweet cherry fruits

**Fig. 8:**
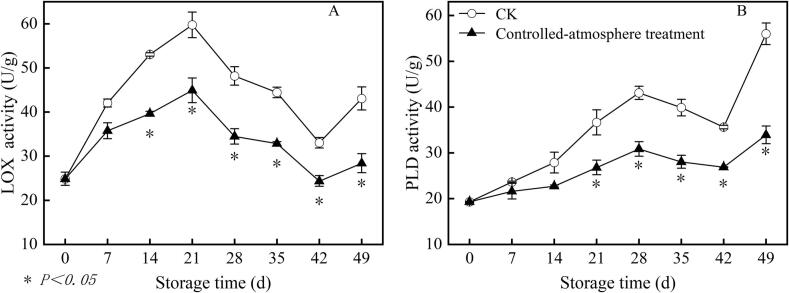
Impact of controlled-atmosphere treatment on LOX (A) and PLD (B) activities of sweet cherry fruits. Data are represented as mean ± SE (n = 3). * indicate significant differences at *P < 0.05*, according to the t-test.

As storage time extended, the LOX activity of sweet cherries peaked on the day 21 of storage (Fig. 8A). The controlled-atmosphere group exhibited a 27.32 % lower LOX activity of than that in the CK group from day 14 to the conclusion of storage (*P < 0.05*).

Fruits in the CK group had higher PLD activity during storage than those in the controlled-atmosphere group (Fig. 8B). The controlled-atmosphere group showed a 30.67 % lower PLD activity compared to that in the CK group from day 21 to the conclusion of storage (*P < 0.05*).

### 3.8 Effects of controlled-atmosphere treatment on the relative expression levels of *PaLOX5* and *PaPLD1* in sweet cherry fruits

**Fig. 9:**
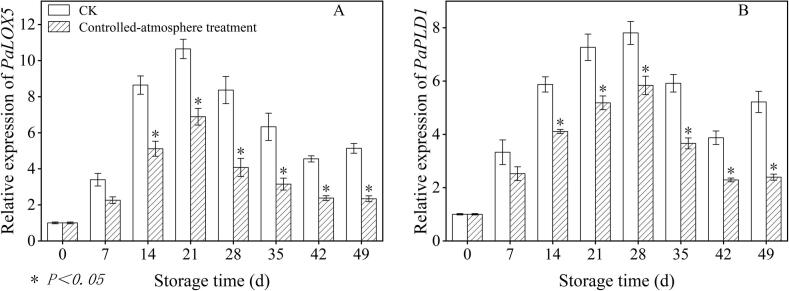
Impact of controlled-atmosphere treatment on the relative expression levels of *PaLOX5* (A) and *PaPLD1* (B) in sweet cherry fruits. Data are denoted as mean ± SE (n = 3). * indicate significant differences at *P < 0.05*, according to the t-test.

The relative expression levels of *PaLOX5* in sweet cherry were inhibited by controlled-atmosphere treatment during storage (Fig. 9A). The controlled-atmosphere group exhibited 45.19 % lower *PaLOX5* relative expression compared to that in the CK group from day 14 until the end of storage (*P < 0.05*).

Over the course of extended storage, the relative expression levels of *PaPLD1* in sweet cherry first peaked on the day 28 of storage (Fig. 9B). In comparison with the CK group, the controlled-atmosphere group showed 34.72 % lower relative *PaPLD1* expression from day 14 to the end of storage (*P < 0.05*).

### 3.9 Effects of controlled-atmosphere treatment on the saturated and unsaturated fatty acid contents in sweet cherry fruits

**Fig. 10:**
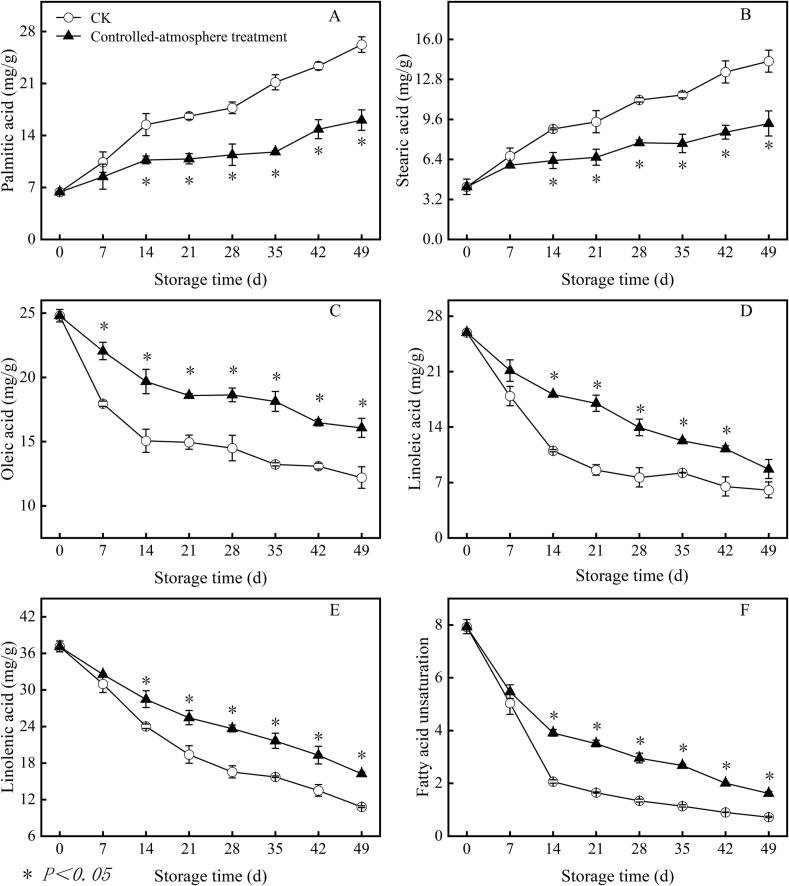
Impact of controlled-atmosphere treatment on the content of palmitic acid (A), stearic acid (B), oleic acid (C), linoleic acid (D), linolenic acid (E) and fatty acid unsaturation (F) in sweet cherry fruits. Data are expressed as mean ± SE (n = 3). * indicate significant differences at *P < 0.05*, according to the t-test.

The controlled-atmosphere treatment delayed the accumulation of palmitic and stearic acids (Figs. 10A and B). From the day 14 to the conclusion of storage, in the controlled-atmosphere group, the contents of stearic acid and palmitic acid were 32.81 % and 37.28 % lower than those in the CK group, respectively (*P < 0.05*).

During storage, the reduction in the oleic, linoleic, and linolenic acid contents was inhibited by controlled-atmosphere treatment (Figs. 10C, D, and E). The controlled-atmosphere group showed a 28.43 % higher content of oleic acid compared to that in the CK group from day 7 to the conclusion of storage (*P < 0.05*). The controlled-atmosphere group showed a 73.27 % greater linoleic acid concentration compared to that in the CK group between days 14 and 42 of storage (*P < 0.05*). The controlled-atmosphere group exhibited 34.69 % higher content of linolenic acid compared to that in the CK group from the day 14 until the end of storage (*P < 0.05*).

The controlled-atmosphere treatment mitigated the decline in fatty acid unsaturation, with levels 113.85 % higher than the CK group from day 14 to the conclusion of storage (*P < 0.05*) (Fig. 10F).

### 3.10 PCA and correlation analysis

**Fig. 11:**
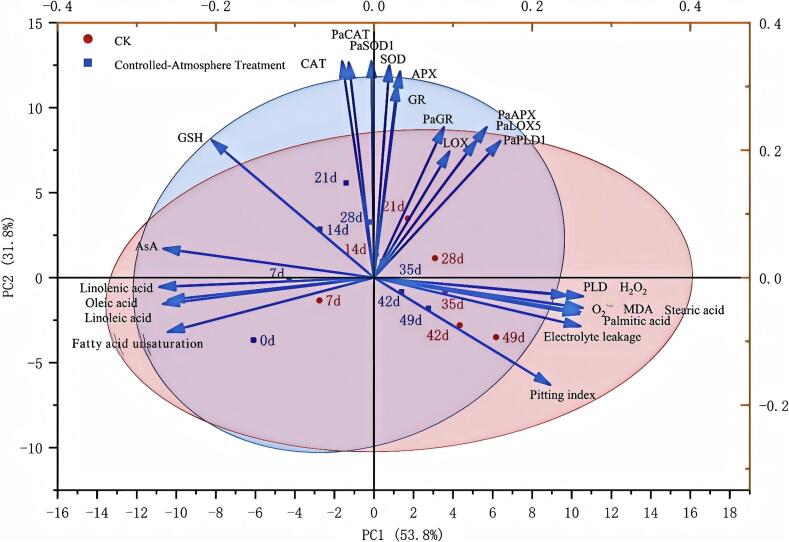
Principal component analysis of controlled-atmosphere treatment on sweet cherry fruits.

PCA was carried out to examine the relevant indicators of sweet cherry in both the controlled atmosphere and the CK groups (Fig. 11). With a cumulative contribution rate of 85.6 %, the contribution rates of principal components 1 (PC1) and 2 (PC2) were 53.8 % and 31.8 %, respectively. The indicators of the CK group were found in the first quadrant over days 14–28, whereas those of the controlled-atmosphere group were dispersed in the second quadrant. The CK group and the controlled-atmosphere group differed significantly.

**Fig. 12:**
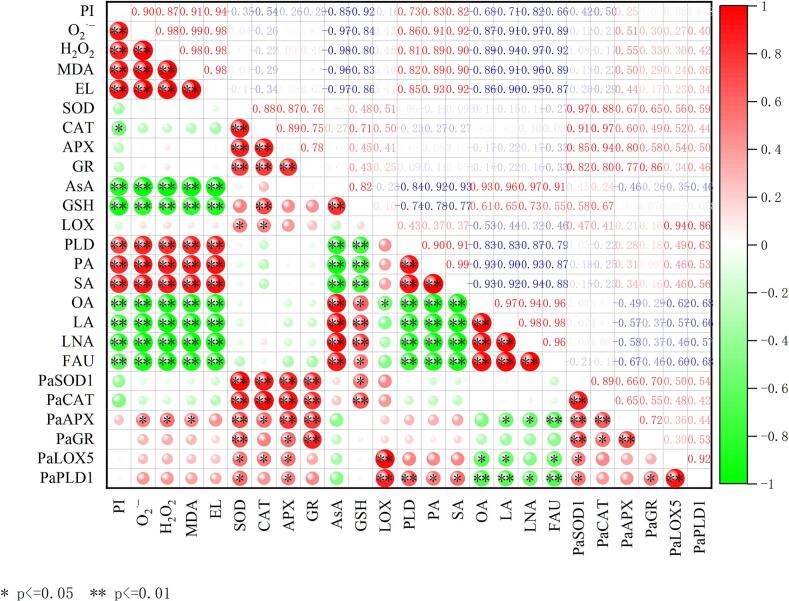
Correlation analysis between antioxidant ability, membrane lipid metabolism parameters and pitting index indices of controlled-atmosphere treatment on sweet cherry fruits. PI indicates pitting index; EL indicates electrolyte leakage; PA indicates palmitic acid; SA indicates stearic acid; OA indicates oleic acid; LA indicates linoleic acid; LNA indicates linolenic acid; and FAU indicates fatty acid unsaturation. Red indicates positive correlation and negative correlation is shown in green. *P<0.05, **P<0.01. (For interpretation of the references to colour in this figure legend, the reader is referred to the web version of this article.)

Correlation analysis exhibited that there was a positive correlation between pitting index, the contents of MDA, H_2_O_2_, O_2_^.-^, and electrolyte leakage in the CK (Fig. 12)(P < 0.05), and a negative correlation between pitting index and activities of APX, SOD, CAT, LOX, GR, AsA, and GSH. Meanwhile, the pitting index showed significant positive correlations with PLD, palmitic, and stearic acids in the CK (*P < 0.05*), while it was significantly negatively associated with oleic, linoleic, linolenic acid, and fatty acid unsaturation (*P < 0.05*). The correlation between ROS and fatty acid unsaturation was significantly negative, suggesting that excessive ROS attacked the cell membranes, further causing oxidative damage.

## 4 Discussion

Sweet cherry has delicate flesh, thin skin, and juicy fruit, making it highly perishable at ambient temperature. Low-temperature storage can effectively extend the shelf life of sweet cherry, but as storage time extends, post-harvest physiological disorders are likely to occur, resulting in fruit surface pitting (Wang et al., 2019; Zhi & Dong, 2018). In the current study, controlled-atmosphere treatment significantly inhibited the increase in pitting rate, pitting index, and decay rate of sweet cherry fruits (Fig. 1, Fig. 2). This is in line with the findings of Kappel et al. on ‘Bing’ sweet cherry (Kappel et al., 2002). The possible mechanism lies in the fact that controlled-atmosphere treatment may trigger the internal defence system of the fruits, enhancing its ability to cope with low-temperature oxidative stress. However, some reports have shown that controlled-atmosphere treatment has no effect on surface pitting of sweet cherries (Karagiannis et al., 2018). This may be attributed to the differences in the ratios of O_2_ and CO_2_ in the controlled-atmosphere environment, as well as the significant variations in the sensitivity and response mechanisms of different varieties to controlled-atmosphere treatment. Furthermore, controlled-atmosphere treatment effectively preserved fruit firmness, C, L*, h°, SSC, and TA content (Fig. 3), thereby maintaining the storage quality of sweet cherry fruits. These results were consistent with Neira-Ojeda et al. (2025) and Martignetti et al. (2019). These effects may be ascribed to the precise regulation of the gaseous composition within the storage environment. By reducing O_2_ levels and elevating CO_2_ concentrations, controlled-atmosphere storage effectively slowed down the aerobic metabolism of sweet cherries. This metabolic slowdown reduced the consumption of essential nutrients and energy reserves, thereby alleviating physiological disorders in the fruits and delaying the emergence of spoilage-related symptoms.

Low-temperature stress leads to an imbalance in cell redox; thus, ROS accumulate continuously. Excessive ROS attack the cell membrane, causing oxidative damage and loss of membrane function, leading to fruit surface pitting and deterioration of fruit quality (Gogoi et al., 2024). In the present study, the pitting index was significantly positively correlated with O_2_^.-^, H_2_O_2_, MDA content, and electrolyte leakage (Fig. 12) (*P < 0.05*). This further confirmed that ROS-induced oxidative damage to cell membranes is a core inducer of sweet cherry pitting disorder. Beyond ROS oxidative damage, abnormal cell membrane metabolism is another core inducer of pitting. The cell membrane is the main site of physiological changes (He et al., 2019). The primary components of cell membranes, fatty acids and phospholipids, are essential for preserving cell membrane function (Wang & Fan, 2023). LOX and PLD, key enzymes in membrane lipid metabolism, catalyse the oxidation of unsaturated fatty acids, thus reducing the unsaturation index of fatty acids and causing cell membranes to transform from a tight and orderly liquid crystal state to a loose and disordered gel state, resulting in decreased cell membrane fluidity and fruit surface pitting (Li et al., 2023). The present study showed that the pitting index exhibited significant positive correlations with PLD, palmitic, and stearic acids (saturated fatty acids), while it was significantly negatively correlated with oleic, linoleic, linolenic acids (unsaturated fatty acids), and fatty acid unsaturation (Fig. 12) (*P < 0.05*). This further confirmed that membrane phase transition because of fatty acid changes is another core inducer of sweet cherry pitting disorder.

ROS accumulation and membrane lipid metabolism disorder do not act independently, but form the core regulatory axis of pitting via “bidirectional coupling and dynamic interaction”, during physiological disorders such as surface pitting. As the “initial signal source”, ROS directly oxidizes membrane lipid unsaturated fatty acids to induce lipid peroxidation and abnormally activates PLD and LOX, disrupting membrane lipid synthesis-degradation balance (Bhattacharjee, 2005; Lin et al., 2019). Conversely, membrane lipid metabolism disorder reduces unsaturated fatty acids, increases saturated fatty acids, damages membrane structure and organelles, inhibits ROS-scavenging enzyme activity, and collapses ROS-scavenging capacity (Gill & Tuteja, 2010; Sharma et al., 2012), forming an “ROS accumulation→membrane lipid disorder→further ROS amplification” vicious cycle. This cycle amplifies slight initial disturbances (e.g., low-temperature stress, micro-injury), ultimately causing membrane disintegration and surface pitting. Notably, prior studies on pomegranate aril browning (Shen et al., 2026) and passion fruit (Lin et al., 2025) disorders showed a unidirectional ROS-membrane lipid relationship, without such bidirectional coupling. In this study, PCA provided intuitive evidence for this coupling mechanism: ROS and membrane lipid damage indicators, including saturated fatty acids, MDA, electrolyte leakage and PLD activity, clustered tightly in the fourth quadrant; in contrast, membrane lipid protection indicators (unsaturated fatty acids and fatty acid unsaturation) concentrated in the third quadrant (Fig. 11). This distinct distribution reflected the opposing clustering of “damage indicators” and “protection indicators”, visually supporting the coupling interaction. Consistently, correlation analysis (Fig. 12) offered quantitative proof: ROS indicators (O_2_^.-^ and H_2_O_2_) showed extremely significant positive correlations (*P* < 0.01) with membrane lipid damage indicators, and extremely significant negative correlations (*P* < 0.01) with membrane lipid protection indicators. These results confirmed the close correlation between ROS accumulation and abnormal membrane lipid metabolism in surface pitting, and revealed their “metabolic synergy”.

The oxidative damage by ROS and degradative metabolism of membrane lipids exhibit a mutually reinforcing effect, jointly constructing the “physiological synergy pathway” underlying the occurrence of pitting disorder. A single regulatory strategy targeting only ROS scavenging or membrane lipid protection fails to break their coupled cycle, rendering effective prevention and control of pitting difficult. Thus, simultaneous intervention in ROS metabolism and membrane lipid balance is essential to fundamentally sever the physiological chain driving pitting occurrence. Reportedly, enhancing the activities of antioxidant enzymes (SOD, CAT, APX, and GR) and levels of antioxidant substances (ASA and GSH) is conducive to the efficient ROS scavenging (Pereira et al., 2023; Singh & Prasad, 2024). Reducing the activities of LOX and PLD can regulate fatty acid composition (increased unsaturated fatty acid and decreased saturated fatty acid contents) ultimately achieving the elevation of fatty acid unsaturation and the protection of membrane structure (Li et al., 2023; Wang & Fan, 2023). In this study, correlation analysis of two antioxidants (ASA; GSH) provided direct evidence for the controlled-atmosphere-mediated coupling regulatory mechanism: they displayed an extremely significant negative correlation (*P* < 0.01) with oxidative damage indicators (O_2_^.-^; H_2_O_2_; MDA; electrolyte leakage) and membrane lipid damage indicators (PLD activity; palmitic acid; stearic acid), while showing a significant positive correlation (*P* < 0.05) with membrane lipid protection indicators (oleic acid; linoleic acid; linolenic acid; fatty acid unsaturation degree) (Fig. 12).

Furthermore, the inhibitory effect of controlled-atmosphere treatment on pitting disorder directly confirmed the core regulatory value of this coupling mechanism, which could be specifically achieved through two synergistic targets: First target (ROS regulation target): Low O_2_ combined with high CO_2_ could directly reduce ROS production by alleviating oxidative stress; meanwhile, this treatment enhanced the activity and gene expression of antioxidant enzymes and increased the content of antioxidant substances (Fig. 5, Fig. 6, Fig. 7), thereby boosting ROS-scavenging capacity. Ultimately, it effectively delayed membrane lipid oxidation, maintained membrane structural integrity, and further blocked the key link initiated by ROS in the physiological chain of pitting disorder (Fig. 4). Second target (Membrane lipid metabolism target): Controlled-atmosphere treatment synchronously downregulated the activity and gene expression of LOX and PLD (Fig. 8, Fig. 9), suppressed membrane lipid degradation and fatty acid peroxidation to consolidate membrane structure and function (Fig. 10). Critically, the stabilized membrane structure maintained the activity of antioxidant enzymes, which further restrained ROS accumulation. It formed a positive feedback loop with first target to interrupt the ROS-membrane lipid cycle, thereby slowing membrane lipid peroxidation and reducing surface pitting. Currently, the core strategy behind controlled-atmosphere preservation for fruits such as jujubes (Li et al., 2023) and lychees (Ali et al., 2016) mainly focuses on reactive oxygen species, with little attention to membrane lipid metabolism. Meanwhile, reports on controlled atmosphere of sweet cherries also focused only on browning enzymes and microbial population changes (Serradilla et al., 2013; Yang et al., 2019).

Based on the characteristics of the “ROS-membrane lipid coupling cycle” in sweet cherries, this study proposes a synergistic strategy of “low oxygen+high CO_2_ control ROS production+stabilize membrane lipids+maintain enzyme system to ensure scavenging ROS”. This strategy is more in line with the multi-link pathogenesis of pitting metabolic disorder compared to the single regulation mode for fruits such as prune (Zhang et al., 2025). However, this study is limited to the “optimal controlled-atmosphere condition (3% O₂+10% CO₂)” for refrigerated sweet cherries, which entailed two constraints: (1) The dominant role and contribution of O_2_ and CO_2_ in the aforementioned synergistic strategy remained unclear; (2) the regulatory thresholds of these two parameters were undefined (failing to evaluate pitting-related changes when concentrations deviate), leaving the core controlled-atmosphere thresholds for anti-pitting unknown. Therefore, future research will expand the O_2_ and CO_2_ concentration ranges, integrate key indicators (e.g., pitting-related pathway enzymes), and verify their effects on sweet cherry storage quality. This will not only refine the theoretical details of the proposed synergistic strategy but also will provide solid practical support for the “dual-target synergistic intervention” in the precise regulation of physiological pitting disorder in fruits.

## 5 Conclusions

The controlled-atmosphere treatment (3% O_2_ + 10% CO_2_ + 87 % N_2_) alleviated surface pitting and maintained fruit quality in sweet cherry. The core mechanism lies in the innovative strategy of “ROS+membrane lipid metabolism” dual-target synergistic regulation-realized through dual-pathway gene-metabolism cascade regulation: it realized dual gene-metabolism pathway regulation-targeting antioxidant enzyme genes to enhance activity, increase antioxidant substances content, and scavenge excess ROS, while first regulating membrane lipid metabolism key enzyme genes to optimize metabolism and maintain cell membrane integrity; their synergy matched the multi-link pathogenesis of pitting disorder. This study provides “gene-metabolism” theoretical support for controlled-atmosphere technology, with broad application prospects.

## CRediT authorship contribution statement

**Qifeng Zhao:** Writing – original draft, Investigation, Formal analysis, Data curation. **Yingjian Qi:** Writing – original draft, Investigation, Formal analysis, Data curation. **Feng Wang:** Writing – review & editing, Supervision, Methodology. **Haixia Yang:** Data curation. **Qingzhen Yang:** Writing – review & editing, Investigation, Funding acquisition, Formal analysis. **Xiaoping Zhang:** Writing – review & editing, Funding acquisition, Formal analysis.

## Declaration of competing interest

The authors declare that they have no known competing financial interests or personal relationships that could have appeared to influence the work reported in this paper.

## Data Availability

Data will be made available on request.
